# Hypoxia-induced angiotensin II by the lactate-chymase-dependent mechanism mediates radioresistance of hypoxic tumor cells

**DOI:** 10.1038/srep42396

**Published:** 2017-02-16

**Authors:** Guozhu Xie, Ying Liu, Qiwei Yao, Rong Zheng, Lanfang Zhang, Jie Lin, Zhaoze Guo, Shasha Du, Chen Ren, Quan Yuan, Yawei Yuan

**Affiliations:** 1 Department of Radiation Oncology, Nanfang Hospital, Southern Medical University, Guangzhou, Guangdong 510515, P.R. China; 2 Department of Radiation Oncology, Teaching Hospital of Fujian Provincial Cancer Hospital, Fuzhou, Fujian 350014, P.R. China; 3 Breast Center, Nanfang Hospital, Southern Medical University, Guangzhou, Guangdong 510515, P.R. China; 4 Jules Stein Eye Institute, David Geffen School of Medicine, University of California, Los Angeles, CA 90095, USA; 5 Department of Radiation Oncology, Cancer Center of Guangzhou Medical University, Guangzhou, Guangdong 510095, P.R. China

## Abstract

The renin-angiotensin system (RAS) is a principal determinant of arterial blood pressure and fluid and electrolyte balance. RAS component dysregulation was recently found in some malignancies and correlated with poor patient outcomes. However, the exact mechanism of local RAS activation in tumors is still unclear. Here, we find that the local angiotensin II predominantly exists in the hypoxic regions of tumor formed by nasopharyngeal carcinoma CNE2 cells and breast cancer MDA-MB-231 cells, where these tumor cells autocrinely produce angiotensin II by a chymase-dependent rather than an angiotensin converting enzyme-dependent mechanism. We further demonstrate in nasopharyngeal carcinoma CNE2 and 5–8F cells that this chymase-dependent effect is mediated by increased levels of lactate, a by-product of glycolytic metabolism. Finally, we show that the enhanced angiotensin II plays an important role in the intracellular accumulation of HIF-1α of hypoxic nasopharyngeal carcinoma cells and mediates the radiation-resistant phenotype of these nasopharyngeal carcinoma cells. Thus, our findings reveal the critical role of hypoxia in producing local angiotensin II by a lactate-chymase-dependent mechanism and highlight the importance of local angiotensin II in regulating radioresistance of hypoxic tumor cells.

The renin-angiotensin system (RAS) is classically known as a circulating or hormonal system that regulates blood pressure and electrolyte and fluid homeostasis^1^. The classical RAS consists of several key components: hepatic-derived precursor angiotensinogen (AGT), renally synthesized renin, pulmonary-bound angiotensin-converting enzyme (ACE), and the physiologically active peptide, angiotensin II (Ang II). Ang II is a key bioactive RAS peptide through binding to its receptors, Ang II type 1 receptor (AT1R) and Ang II type 2 receptor (AT2R); most of the functions are, however, mediated by the AT1R^2^. In addition to the circulating RAS, local tissue RAS has been increasingly found in diverse organ systems, including the kidney, heart, vasculature, pancreas, and adipose tissue^3^. In these tissues, angiotensinogen, renin, ACE and Ang II receptors are invariably locally synthesized and local Ang II functions in an autocrine or paracrine manner and participates in organ homeostasis and the pathogenesis of chronic diseases^3^.

AT1R blockers (ARBs) have been widely used in the treatment of a variety of cardiovascular and chronic kidney diseases. Interestingly, a retrospective study provided evidence that patients with hypertension taking ACE inhibitors and ARBs had a decreased risk of developing some types of cancers^4^. Subsequent studies of animal xenograft models and human malignancies observed the frequent dysregulation of RAS components and the correlation with disease outcomes^1^, indicating an important role for local RAS in modulating tumor biology. However, the exact mechanism of the activation of local RAS in tumors is still unclear.

Hypoxia is a frequent feature of the tumor microenvironment in a broad spectrum of solid tumors, especially for head-neck carcinomas, breast cancers, gliomas and soft tissue sarcomas^5^,^6^,^7^,^8^,^9^,^10^. Hypoxia usually results in resistance of these tumors to radiation therapy through inducing activation of the HIF signaling pathway which enhances DNA double-strand break repair activity, and spurring anaerobic glycolysis, angiogenesis, and survival of tumor cells^11^,^12^,^13^. Previous studies have shown that Ang II can be locally produced in hypoxic somatic tissues and that this Ang II overproduction plays an important role in the development of chronic disease, such as atherosclerosis^14^, diabetic nephropathy^15^, and retinopathy^16^. However, whether intratumoral hypoxia induces the generation of local Ang II and their role in regulating radioresistance of hypoxic tumor cells are still unknown. Considering that nasopharngeal carcinomas and breast cancers are important models as hypoxic tumors, nasopharngeal carcinoma and breast cancer cell lines were used herein to demonstrate the impact of hypoxia on Ang II formation in tumor cells. Our study reveals the critical role of hypoxia in producing local Ang II by a lactate-chymase dependent mechanism and highlights the important role of local Ang II in regulating radioresistance of tumor cells in the hypoxic microenvironment.

## Results

### Local Ang II is activated predominantly in a hypoxic tumor microenvironment

**Figure 1:**
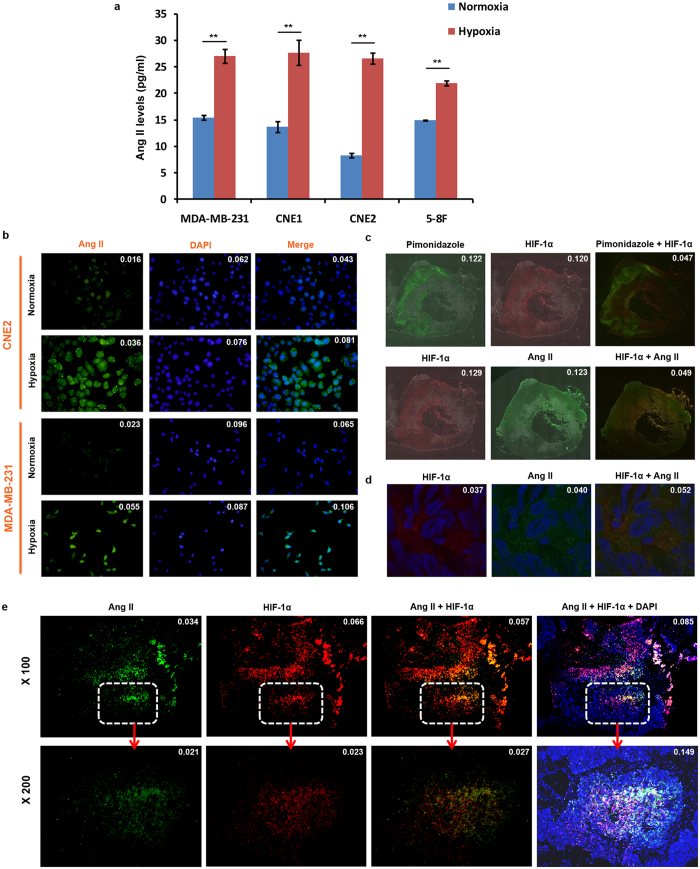
Local RAS predominantly localizes in the hypoxic tumor microenvironment. **(a)** Expression analysis by ELISA (enzyme linked immunosorbent assay) of Ang II in the supernatant of hypoxic tumor cells (n = 3). **(b)** Ang II expression detection by IF (immunofluorescence) in *in vitro* tumor cells. **(c,d)** Ang II expression detection by confocal laser scanning microscopy in xenografted tumors in mice. **(e)** Ang II expression detection by IF in human nasopharyngeal carcinoma specimens. **P < 0.01, using a two-tailed, unpaired t-test. All experiments were performed at least thrice.

To determine whether hypoxia contributes to the generation of Ang II in tumor cells, we first measured the Ang II levels in the supernatant of *in vitro* cultured tumor cells from CNE1, CNE2 and 5–8F nasopharyngeal carcinoma cells and MDA-MB-231 breast cancer cells, both of which display predominant hypoxia in their solid tumors^17^,^18^. Analysis of an enzyme-linked immunosorbent assay (ELISA) showed that only low levels of Ang II were detected in the supernatant of tumors cells from all these cell lines when cultured under normoxic (20% O_2_) conditions. However, significantly higher Ang II levels were detected when tumor cells were cultured in hypoxic conditions (containing 1% O_2_; Fig. 1a). Furthermore, using immunofluorescence (IF) analysis, we detected low levels of expression of Ang II in the cytoplasm of normoxic-cultured CNE2 and MDA-MB-231 cells; however, the expression of Ang II was significantly increased in hypoxic-cultured cells (Fig. 1b). Importantly, in xenografted tumors in mice, we found that Ang II predominantly accumulated in the hypoxic regions of the tumors, which were identified using an exogenous hypoxia marker, pimonidazole, and an endogenous hypoxia marker, HIF-1α (Fig. 1c). Co-localization of Ang II and HIF-1α proteins in the cytoplasm of hypoxic tumor cells was also detectable in confocal laser scanning microscopy (Fig. 1d). To further confirm this observation, we determined the expression levels of Ang II and HIF-1α in 13 human nasopharyngeal carcinoma specimens. We found that Ang II was consistently present in HIF-1α-expressing regions of human nasopharyngeal carcinoma specimens (Fig. 1e). These results, thus, verify the possibility that hypoxia greatly induces the generation of Ang II by the tumor cells themselves in hypoxic tumor regions.

**Figure 2:**
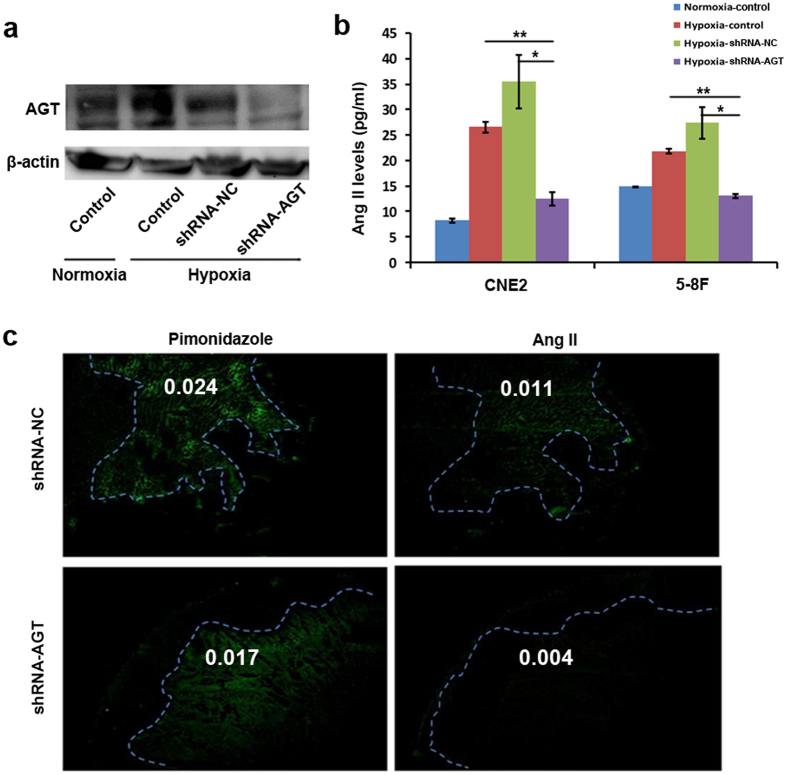
Ang II presented as an autocrine product of tumor cells in hypoxic regions. **(a)** The angiotensinogen (AGT) gene was stably silenced in CNE2 tumor cells, as demonstrated by Western blot. (**b)** AGT-silence considerably reduced Ang II levels in the supernatant of CNE2 and 5–8F tumor cells as detected by ELISA. (**c)** AGT-silence attenuated Ang II levels in pimonidazole-positive hypoxic regions of *in vivo* tumors formed by CNE2 cells. *P < 0.05, **P < 0.01.

To further confirm whether the Ang II detected in hypoxic tumor tissues is an autocrine product of tumor cells, the expression of AGT, a precursor of angiotensin II, was stably silenced in tumor cells by lentiviral vector-mediated short hairpin RNA (shRNA) (Fig. 2a and Supplementary Figure S1). The inhibition of AGT expression greatly decreased Ang II levels in the supernatant of the hypoxic-cultured tumor cells (Fig. 2b). Furthermore, the tumor cells were subcutaneously injected into BALB/c nude mice. The tumors in mice were harvested and analyzed for the presence of Ang II by IF. Interestingly, Ang II was markedly attenuated in pimonidazole-positive regions of the tumors formed by AGT-inhibited tumor cells (Fig. 2c). These findings further support the model that Ang II detected in hypoxic tumor regions is predominantly produced by the tumor cells themselves.

### Hypoxic tumor cells produce Ang II via a chymase-dependent mechanism

**Figure 3:**
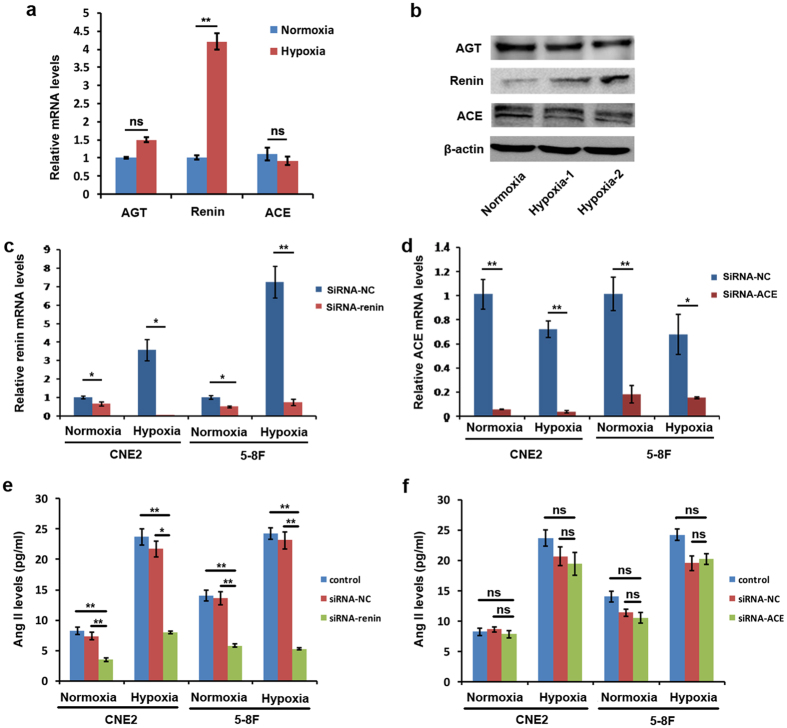
The generation of Ang II in hypoxic tumor cells is independent of ACE-mediated pathway. (**a**) The gene expression analysis of CNE2 cells showed that only renin displayed remarkably enhanced expression in the hypoxic condition, whereas AGT (angiotensinogen) and ACE (angiotensin-converting enzyme) had similar expression levels in the hypoxic condition compared with the normoxic condition. (**b**) The expression of RAS components in CNE2 cells was also further verified by Western blot analysis; hypoxia-1, 5% O2 condition; hypoxia-2, 1% O_2_ condition. (**c**) The expression of renin in CNE2 cells and 5–8F cells was significantly inhibited by siRNA-renin as detected by quantitative RT-PCR. (**d**) The expression of ACE in CNE2 cells and 5–8F cells was significantly inhibited by siRNA-ACE as detected by quantitative RT-PCR. (**e**)The suppressed expression of renin by siRNA led to the decreased Ang II levels in both normoxic and hypoxic tumor cells. (**f**) The suppressed expression of ACE by siRNA did not significantly decreased Ang II levels in both normoxic and hypoxic tumor cells.

Our data showed that local RAS is predominantly activated in hypoxic tumor regions. Next, we sought to identify the mechanism for generation of Ang II under hypoxic conditions. We assessed the expression of classical RAS components (AGT, renin, and ACE) in hypoxic CNE2 cells. Unexpectedly, the gene expression analysis showed that only renin displayed remarkably enhanced expression in the hypoxic condition, whereas AGT and ACE had similar expression levels in the hypoxic condition compared with the normoxic condition (Fig. 3a), which was also further verified by Western blot analysis (Fig. 3b and Supplementary Figure S2). Importantly, we found that the suppression of renin expression in CNE2 and 5–8F cells through short-interfering RNA (siRNA)-mediated silencing led to the decreased Ang II levels in hypoxic CNE2 and 5–8F cells (Fig. 3c,e). Notably, the suppression of renin also decreased Ang II levels in these cells under normoxic condition, indicating renin is probably essential in the generation of Ang II in these cells and this impact of renin on Ang II levels may be independent of oxygen content. On the other hand, the expression of ACE in CNE2 cells and 5–8F cells was also significantly inhibited by siRNA-ACE in both normoxic and hypoxic conditions (Fig. 3d), but the suppressed expression of ACE did not significantly decreased Ang II levels in both normoxic and hypoxic cells (Fig. 3f). These results suggest that the enhanced levels of Ang II in hypoxic tumor cells are probably not mediated by the canonical angiotensinogen-rennin-ACE pathway.

**Figure 4:**
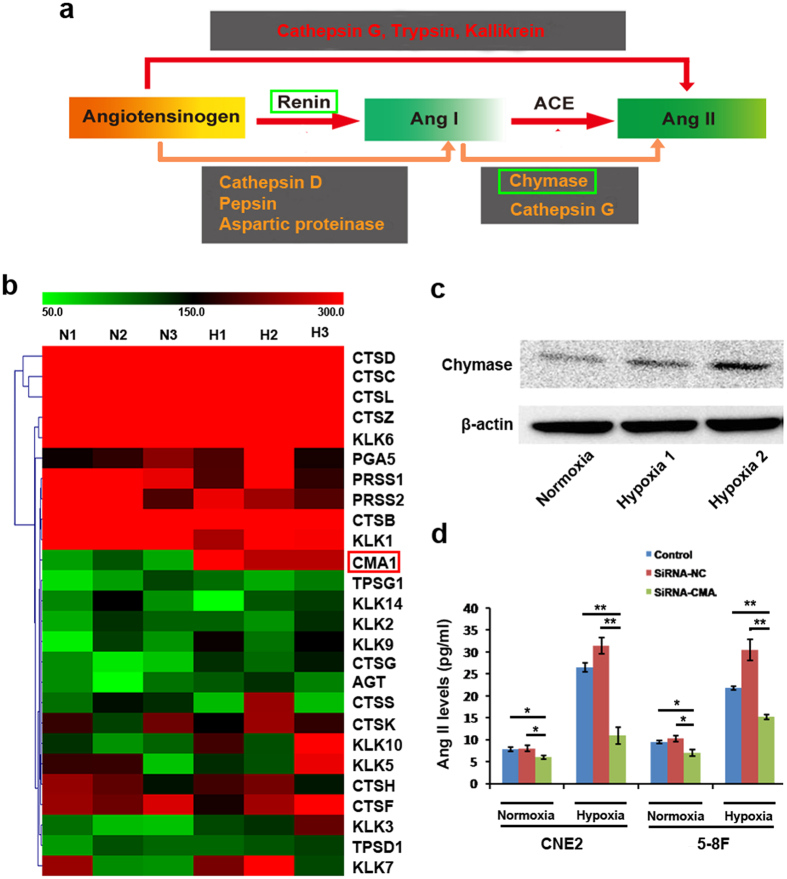
The role of chymase in the generation of Ang II in hypoxic tumor cells. **(a)** Ang II generation by ACE-dependent or independent pathways in a variety of human organs in physiologic and pathophysiologic conditions. (**b**) Gene array analysis revealed that CMA1 expression was significantly upregulated in all hypoxic CNE2 cells; N1, N2 and N3 referred to triplicates in normoxic condition; H1, H2 and H3 referred to triplicates in hypoxic condition. (**c)** CMA1 expression in CNE2 cells was confirmed by protein analysis; hypoxia-1, 5% O2 condition; hypoxia-2, 1% O2 condition. **(d)** ELISA analysis showed an obvious decrease of Ang II levels in hypoxic cells when the expression of chymase was suppressed by shRNA. *P < 0.05; **P < 0.01; ns, no significance. ACE, angiotensin-converting enzyme; Ang I, angiotensin I; Ang II, angiotensin II. CMA, chymase; ELISA, enzyme linked immunosorbent assay.

Previous studies provided evidence for the presence of diverse proteinases responsible for the local production of bioactive Ang II, acting through ACE-independent pathways (Fig. 4a), in a variety of human organs, such as heart, arteries, and kidneys, and involved in the regulation of normal and pathological physiological processes^19^,^20^. Therefore, we used gene expression microarray analysis to evaluate the expression of the previously-reported proteinases involved in Ang II-formation. The mRNA expression profiling analysis revealed that transcripts encoding chymase were significantly increased in all hypoxic CNE2 cell samples (Fig. 4b), which was further confirmed by protein analysis (Fig. 4c and Supplementary Figure S3). Chymase has been reported as a crucial enzyme of the ACE-independent pathway of Ang II generation, through the conversion of Ang I to Ang II, in human and animal heart tissues^21^,^22^. This process appears to predominantly take place in hypoxic or ischemic heart or blood vessels *in vivo* and is involved in the pathological changes therein^23^. Therefore, we concluded that chymase most probably plays an identically important role in enhancing Ang II levels in hypoxic tumor cells. To address this proposed role of chymase, we inhibited its expression in CNE2 and 5–8F cells by lentiviral vector-mediated shRNA. We found that the suppression of chymase expression by shRNA led to significantly decreased Ang II levels in the supernatant of hypoxic cells almost to the levels detected in normoxic tumor cell culture supernatant (Fig. 4d). These findings reveal that chymase-mediated Ang II generation is alternative to the ACE-dependent Ang II-formation in the hypoxic microenvironment of tumors

### Tumor-derived lactate is responsible for Ang II production in hypoxic tumor cells

**Figure 5:**
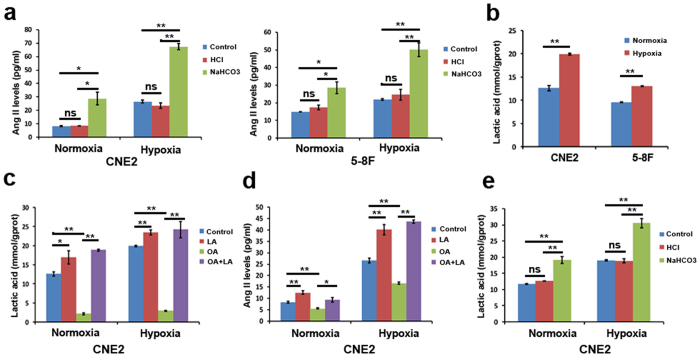
The role of lactate in Ang II generation in hypoxic tumor cells. **(a)** The impact of acidic pH on Ang II (angiotensin II) levels in hypoxic tumor cells. (**b)** Lactate levels in cytoplasm detected by Lactate Assay in hypoxic- and normoxic-cultured tumor cells. (**c**) Lactate levels in different conditions (LA, addition of lactate; OA, pretreated by oxamic acid; LA + OA, pretreated by oxamic acid and addition of lactate). (**d)** Ang II levels in different conditions. **(e)** The influence on lactate levels of the acidification with HCl or alkalinization with NaHCO_3_. *P < 0.05; **P < 0.01; ns, no significance.

Previous data indicated that an acidic pH in the ischemic heart could increase chymase activities, which favored Ang II production from chymase under acidic conditions^24^,^25^. To determine whether an acidic pH was responsible for the chymase-mediated generation of Ang II in hypoxic tumor cells, we adjusted the pH of the conditioned medium from normoxic-cultured CNE2 and 5–8F cells to the level of hypoxic-conditioned medium by adding 1% hydrochloric acid (HCl) (Supplementary Figure S4a). We found that acidification with HCl did not significantly increase the Ang II levels in normoxic-conditioned medium (Fig. 5a). Furthermore, we also adjusted the pH of the hypoxic-conditioned medium of CNE2 and 5–8F cells to the level of normoxic-conditioned medium by adding 5% sodium bicarbonate (NaHCO_3_) during hypoxia. Interestingly, we found that the alkalinization not only markedly enhanced the Ang II levels in the hypoxic-conditioned medium of CNE2 and 5–8F cells but also increased the Ang II levels in the normoxic-conditioned medium (Fig. 5a). These findings suggest that the acidic pH may not be responsible for the chymase-mediated Ang II generation in hypoxic tumor cells.

Lactate is one of the most abundant products of glycolysis and its accumulation acidifies pH in the hypoxic tumor microenvironment^26^. Therefore, we next explored whether lactate played an important role in Ang II generation in hypoxic tumor cells. Results of a lactate assay showed that hypoxic-cultured CNE2 and 5–8F cells produced significantly more lactate in the cytoplasm than the normoxic-cultured cells (Fig. 5b). Interestingly, we found that the addition of 5 mM lactate significantly increased the Ang II levels in both the normoxic- and hypoxic-conditioned supernatant media of CNE2 and 5–8F cells (Fig. 5c,d and Supplementary Figure S4b,c). Conversely, the Ang II levels were markedly decreased when tumor cells were pretreated with oxamic acid (OA), an inhibitor of lactate dehydrogenase that blocks the lactate production under both hypoxic and normoxic conditions (Fig. 5c,d and Supplementary Figure S4b,c). The addition of exogenous lactate to OA-pretreated tumor cell culture restored Ang II levels in normoxic and hypoxic cultures (Fig. 5c,d and Supplementary Figure S4b,c). Interestingly, we found that adding NaHCO_3_ to tumor cells under hypoxia or normoxia also remarkably increased lactate production, whereas the addition of HCl did not (Fig. 5e and Supplementary Figure S4d). These findings strongly indicate that it is the lactate, not the drop in pH, responsible for the enhanced Ang II levels in *in vitro* hypoxic tumor cells.

### Tumor-derived lactate induces the expression of chymase in tumor cells

**Figure 6:**
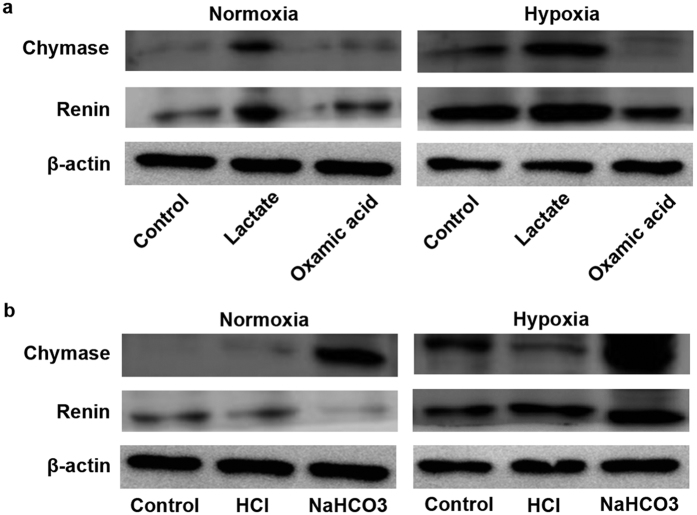
The role of lactate or pH values on renin-chymase-mediated Ang II generation. (**a**) Exogenous lactate increased the expression of chymase and rennin in CNE2 cells, whereas the pretreatment with oxamic acid (OA) suppressed the expression of chymase and renin under normoxic as well as hypoxic conditions. (**b**) The impact of the acidification with HCl or alkalinization with NaHCO_3_ on the expression of chymase and rennin in CNE2 cells.

To further address the role of lactate in chymase-mediated Ang II generation (Fig. 4a), we evaluated whether lactate impacts the expression of chymase. We found that addition of lactate increased the expression of chymase in CNE2 cells under not only the normoxic, but also the hypoxic condition (Fig. 6a and Supplementary Figure S5a). Conversely, pretreatment with OA obviously suppressed the expression of chymase in normoxic and hypoxic CNE2 cells (Fig. 6a). Interestingly, in line with the role of alkalinization in inducing lactate production in tumor cells, addition of NaHCO_3_ to CNE2 cells led to the increased expression of chymase compared with the expression in the control and HCl-treated group, both in normoxic and hypoxic conditions (Fig. 6b and Supplementary Figure S5b). In addition, we found that lactate production is also involved in the expression of renin in CNE2 cells (Fig. 6a and Supplementary Figure S5a). The addition of exogenous lactate increased the expression of renin in normoxic and hypoxic CNE2 cells, and the pretreatment of cells with OA suppressed the expression of renin in these cells (Fig. 6a and Supplementary Figure S5a). Additionally, we found that the addition of NaHCO_3_ to CNE2 cells moderately increased the expression of renin in the hypoxic, but not the normoxic condition (Fig. 6b and Supplementary Figure S5b). Together, these findings suggest that the enhanced lactate levels might be critical for the expression of chymase in hypoxic tumor cells.

### Ang II contributes to the accumulation of HIF-1α protein in hypoxic tumor cells

**Figure 7:**
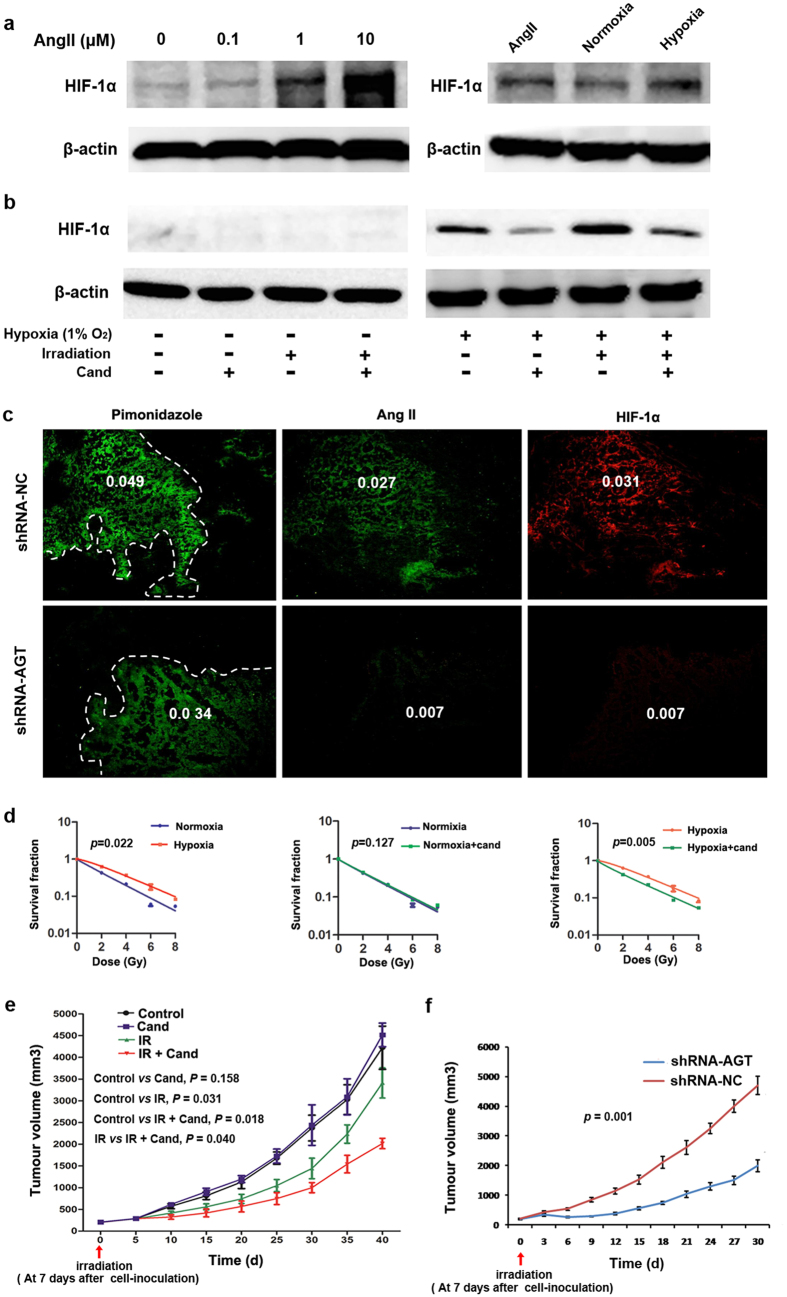
Ang II is involved in the accumulation of HIF-1α in the tumor hypoxic microenvironment. **(a)** Ang II increased HIF-1α protein levels of CNE2 cells in a dose-dependent manner under normoxic conditions, and hypoxia exposure increased HIF-1α accumulation in tumor cells. (**b**) Candesartan remarkably inhibited HIF-1α protein levels of hypoxic (and even irradiated) CNE2 tumor cells. (**c)** Stable suppression of AGT expression in CNE2 cells greatly attenuated the HIF-1α accumulation in pimonidazole-positive hypoxic regions of xenografted tumors in mice. (**d)** Candesartan greatly increased radiosensitivity of hypoxic but not normoxic CNE2 cells. (**e**) Ten-Gy irradiation combined with candesartan treatment significantly reduced CNE2 tumor volumes in mice as compared with irradiation alone; n = 5. (**f)** AGT-silence by lentiviral-AGT-shRNA consistently reduced CNE2 tumor volumes in mice after 10-Gy irradiation, as compared with the negative-control group; n = 5.

Ang II has been shown to contribute to the accumulation of HIF-1α protein in interstitial and vascular smooth muscle cells, thereby participating in renal damage^27^,^28^. Therefore, we sought to demonstrate whether in hypoxic tumor cells Ang II is involved in the accumulation of HIF-1α protein, a key regulator of the hypoxic response. Western blot analyses showed that Ang II increased HIF-1α protein levels of CNE2 cells in a dose-dependent manner under normoxic conditions (Fig. 7a and Supplementary Figure S6a), indicating that Ang II can stimulate HIF-1α accumulation in tumor cells *in vitro*. Exposure to hypoxia is known to increase HIF-1α accumulation in tumor cells^29^, which was also confirmed in our results (Fig. 7a and Supplementary Figure S6b). Notably, candesartan, an ARB, significantly decreased HIF-1α protein levels of hypoxic tumor cells (Fig. 7b and Supplementary Figure S6c). Furthermore, AGT-silencing similarly resulted in reduced expression of HIF-1α protein in hypoxic tumor cells (Supplementary Figure S7a). Stable suppression of AGT expression in CNE2 tumor cells by RNA interference (RNAi) greatly attenuated HIF-1α accumulation in the pimonidazole-positive hypoxic regions of the xenografted tumors formed in nude mice (Fig. 7c). These findings support a role of Ang II as an important regulator involved in the accumulation of HIF-1α in a hypoxic tumor microenvironment.

### Ang II signal blocking reverses the hypoxia-induced radiation resistance of tumor cells

The role of HIF-1α in promoting radiation resistance of hypoxic tumor cells is well established^30^,^31^. Given the important role of Ang II in HIF-1α accumulation in hypoxic tumor cells, we next assessed whether the enhanced Ang II levels contribute to radiation resistance of hypoxic tumor cells. As expected, hypoxia exposure markedly augmented the radioresistance of CNE2 and 5–8F cells (Fig. 7d; Supplementary Figure S7b), as measured by an *in vitro* colony formation assay. Interestingly, the radioresistance of hypoxic CNE2 and 5–8F cells was greatly reversed if these tumor cells were pretreated with candesartan (Fig. 7d, Supplementary Figure S7b), although candesartan did not significantly increase the radiosensitivity of CNE2 and 5–8F cells in the normoxic condition (Fig. 7d, Supplementary Figure S7b). Moreover, using an *in vivo* xenograft model, we found that although candesartan alone could not inhibit CNE2 tumor growth in immunodeficient nude mice, tumors treated with candesartan (administered daily for 3 days before irradiation and 3 days after irradiation) and irradiation (10 Gy) significantly reduced tumor volumes as compared with those treated with irradiation alone (Fig. 7e). In addition, tumors formed by AGT-silenced CNE2 and 5–8F cells displayed consistently reduced tumor volumes after irradiation with 10 Gy, as compared with those formed by cells treated with a negative control shRNA vector (Fig. 7f and Supplementary Figure S7c). These findings suggest that the increased Ang II levels in the tumor microenvironment contribute to the radiation resistance of hypoxic tumor cells, and the inhibition of Ang II signaling leads to reversal of the hypoxia-induced radiation resistance of tumor cells.

## Discussion

The role of local RAS in tumors is becoming increasingly recognized based on the frequent dysregulation of its components in tumors and the correlation with disease outcomes. However, the exact mechanism of the activation of local RAS in tumors has not yet been elucidated. Hypoxia is an important hallmark of many human solid tumors and plays a critical role in tumor biology. Previous studies showed that local Ang II plays an important role in the pathogenesis of chronic diseases, such as atherosclerosis^14^, diabetic nephropathy^15^, and retinopathy^16^, by upregulating GLUT1, HIF-1α, and VEGF expression. It is notable that all these factors are commonly upregulated in hypoxic tumor cells and induce adaptive responses to hypoxia, including glycolysis, angiogenesis, and pH regulation^32^,^33^. Herein, our results found that local Ang II predominantly existed in hypoxic regions of solid tumors, and revealed a lactate-chymase-dependent mechanism responsible for the generationof local Ang II in the tumor microenvironment.

Classical RAS in circulation includes renin-mediated enzymatic catalysis of the conversion of angiotensinogen to Ang I, followed by ACE-mediated cleavage of Ang I to Ang II, and activation of AT1 receptors, which are responsible for RAS biologic actions. In local tissues, except for the classical RAS mechanism, several types of proteinases have been found to be responsible for Ang II formation and are involved in the pathological changes of various organs^34^,^35^,^36^. Interestingly, in this study, we found that Ang II generation in hypoxic tumor cells was independent of ACE expression. Nevertheless, we uncovered a critical role for chymase in Ang II formation in hypoxic tumor cells, and showed that loss of chymase, via silencing of chymase expression, abrogates hypoxia-induced formation of Ang II in tumor cells. Previous studies found that chymase was a major Ang II-forming enzyme by converting Ang I into Ang II in ischemic hearts^37^. Actually, the ischemic heart has lower oxygen content for cardiocytes, akin to the hypoxic status of tumor cells. Additionally, our results showed that the suppression of chymase also led to modestly decreased Ang II levels in the supernatant of normoxic CNE2 cells (Supplemental Fig. 3), indicating chymase has a role in the generation of Ang II in both hypoxic and normoxic tumor cells. Nevertheless, the role of chymase became much more important for hypoxic tumor cells due to the significantly enhanced expression of chymase in hypoxic tumor cells compared with their normoxic cells.

The acidic pH in ischemic hearts has been shown to increase the activity of chymase, which favors Ang II production under acidic conditions^24^,^25^. However, our data show that acidic pH *per se* did not lead to an increase in Ang II expression in normoxic tumor cells; conversely, the alkalinization with NaHCO_3_ surprisingly increased the Ang II levels not only in hypoxic but also normoxic tumor cells. These results cannot be simply explained by the contribution of an acidic pH to chymase-mediated Ang II formation in hypoxic tumor cells. Given that lactate, one of the most abundant products of glycolysis, is the main contributor to the acidic pH of the hypoxic tumor microenvironment^26^, and that glycolysis is the main energy metabolism mechanism in the hypoxic tumor microenvironment^38^, we addressed the role of lactate in Ang II formation in hypoxic tumor cells. We found that lactate indeed played a critical role in Ang II formation in hypoxic tumor cells, and that lactate deprivation mediated by pretreatment of cells with an inhibitor of lactate dehydrogenase decreased Ang II levels in hypoxic tumor cells. Interestingly, we found that alkalinization with NaHCO_3_ remarkably increased lactate production in tumor cells, not only under normoxic but also hypoxic circumstances. To determine whether NaHCO_3_ directly impacted the lactate assay, we detected lactate in cell-free media added with NaHCO_3_, and the result showed that lactate was almost undetectable in the cell-free media with NaHCO_3_, which indicated that NaHCO_3_ *per se* increased lactate production rather than impacted the lactate assay. Actually, Mazzio, E.A. *et.al*. have reported that alkaline extra-cellular pH promoted glycolytic processes and produced more lactate in malignant neuroblastoma N2a cells and they further suggested that the glycolytic effect in N2a cells and pericellular acidic pH formed a negative feedback loop^39^. In our results, NaHCO_3_ increased lactate produce of tumor cells and a possible explanation is that NaHCO_3_ neutralizes the pericellular acidic microenvironment and thus abrogates the negative feedback inhibition of pericellular acidic pH on glycolysis metabolism. The increased lactate from alkalinization would then promote Ang II production in tumor cells upon addition of NaHCO_3_ into the culture system, regardless of the oxygenation status.

Ang II has been shown to induce the expression of HIF-1α during the pathogenesis of chronic diseases^41^. In this study, we found that Ang II promotes HIF-1α expression in hypoxic tumor cells and that Ang II signal-blocking abrogates hypoxia-induced HIF-1α expression, indicating a crucial role for the local RAS in intracellular accumulation of HIF-1α protein in the hypoxic tumor microenvironment. HIF-1α-mediated tumor resistance to radiotherapy is a well-demonstrated effect in various solid tumors^42^. Our results showed that pretreatment with candesartan, an AT1R blocker, reversed the resistance of hypoxic tumor cells to radiation exposure; this effect may correlate with the decreased HIF-1α expression due to Ang II signal-blocking in hypoxic tumor cells (Fig. 7b).

Our results showed that hypoxia contributed to lactate product which may induce an acidic pericellular microenvironment. It is not clear whether pericellular acidic microenvironment affects the resistibility of radiation therapy on solid tumor. Our results found that adjusting the acidic pH of the conditioned medium of hypoxic CNE2 and 5–8F tumor cells remarkably increased lactate production which promoted Ang II generation by inducing expression of chymase and renin. We further provided evidence that it is the increased abundance of lactate, not the drop in pH, responsible for the enhanced Ang II levels in hypoxic tumor cells. Therefore, it was difficult to conclude whether pericellular acidic microenvironment affected the resistibility of radiation therapy on solid tumor; but our results suggested that lactate, at least to some extent, played the role in mediating radioresistance of hypoxic tumor cells through promoting Ang II generation and consequent HIF-1α expression.

In summary, our study found that local Ang II predominantly exists in a hypoxic tumor microenvironment, in which tumor cells autocrinely produce Ang II through a chymase-dependent rather than an ACE-dependent mechanism. Tumor cell-derived lactate, rather than the drop of pH values in hypoxic conditions has an important signaling role in inducing Ang II generation by activating expression of renin and chymase in hypoxic tumor cells. Finally, we revealed that the increased Ang II levels promote the accumulation of HIF-1α in hypoxic tumor cells, and mediates the radiation-resistant phenotype of these cells.

## Materials and Methods

### Cell lines and culture

The MDA-MB-231 breast cancer cells and CNE1, CNE2 and 5–8F nasopharyngeal carcinoma cells were purchased from American Type Culture Collection (ATCC; Manassas, VA, USA) and were maintained in RPMI-1640 medium supplemented with 10% fetal bovine serum at 37 °C in an atmosphere of 5% CO_2_ in a humidified incubator. For hypoxic culture, cells were cultured in an inflatable hypoxia chamber (37 °C; an atmosphere of 5% or 1% O_2_ and 5% CO_2_, balanced with N_2_; and humidified). For normoxic culture, cells were cultured in an atmosphere containing 20% O_2_ (37 °C and an atmosphere of 5% CO_2_ equilibrated with atmospheric O_2_ in a humidified incubator).

### ELISA

Ang II ELISA kits were obtained from Ray Biotech, Inc. (Norcross, GA, USA). The ELISA for Ang II was carried out according to the manufacturer’s instructions. Briefly, the microplate in the kit, which is pre-coated with anti-rabbit secondary antibody, was incubated with an anti-Ang II antibody, under such conditions that both biotinylated Ang II peptide and a peptide standard or targeted peptide in samples interact competitively with the Ang II antibody. Unbound biotinylated Ang II peptide was then allowed to interact with streptavidin-horseradish peroxidase (SA-HRP), which catalyzes a color development reaction. The intensity of the colorimetric signal is directly proportional to the amount of the biotinylated peptide-SA-HRP complex and inversely proportional to the amount of the Ang II peptide in the standard or samples. A standard curve of known concentration of Ang II peptide was established, and the concentration of Ang II peptide in the samples was then calculated by interpolation onto the standard curve. To ensure equal number of cells in different groups during ELISA, different groups of tumor cells were respectively seeded at a density of 200,000 cells/well in 1 mL medium in 24-well plates.

### Determination of lactate concentrations

The lactate concentration was measured using a Lactate Assay Kit (Jiancheng; Nanjing, China) according to the manufacturer’s instructions. The mean values and standard error of the mean (SEM) of the lactate concentration were calculated for each condition. All Lactic Acid assays were normalized to total number of 1 × 10^6^ cells at time of assay.

### Immunofluorescence

Initially, 5-μm frozen tissue sections were fixed with cold acetone for 15 minutes at 4 °C and then washed thrice, five minutes each time, with phosphate-buffered saline. Slides were blocked for one hour in 5% bovine serum albumin at room temperature. Primary antibodies were incubated overnight at 4 °C. Following the washes, slides were incubated for one hour at room temperature with the appropriate secondary antibody and 4′,6-diamidino-2-phenylindole (DAPI) counterstain. To reduce autofluorescence, slides were subsequently incubated in 0.3 M glycine for 10 minutes and then mounted in Hydromount aqueous mounting medium (Fisher Scientific). Images were acquired with a fluorescence confocal microscope (Olympus FV10i) using Olympus FV10-ASW software. Antibodies for Immunofluorescence were listed in Supplementary Table 1.

### Western blotting

Whole cell lysates were harvested and Western blotting was conducted as described previously^43^. Primary antibodies are listed in Supplementary Table 1. Blots were visualized using an ECL detection kit (Millipore). Antibodies for Western blotting were listed in Supplementary Table 1.

### Colony formation assay

Colony formation assay was performed to determine radiosensitivity, as described previously^44^. Cells were plated in 6-well plates and allowed to adhere overnight before exposure to radiation at the indicated doses at a dose rate of 500 cGy/min, using 6-MV X-rays generated by a linear accelerator (Varian 2300EX; Varian, Palo Alto, CA). After incubation for 10–14 days, the cells were stained with 0.5% crystal violet in methanol. Then, the colonies (clusters of >50 cells) were counted under a microscope. Survival data from different experiments were pooled, after which, the survival curves were fitted and analyzed using a single-hit, multi-target model.

During colony forming assays for hypoxic cells, tumor cells were exposed to hypoxic condition (1% O_2_) for 8 hours before irradiation and maintained for 16 hours after irradiation. To determine the effect of candesartan on radiosensitivity of tumor cells, candesartan was administrated 12 hours before irradiation and tumor cells were continuously exposed to candesartan for another 24 hours after irradiation.

### DNA microarray analysis

Total RNA from each sample was quantified by the NanoDrop ND-1000, and RNA integrity was assessed by standard denaturing agarose gel electrophoresis. The total RNA of each sample was used for labeling and array hybridization as follows: (step 1) reverse transcription with an Invitrogen Superscript ds-cDNA synthesis kit; (step 2) ds-cDNA labeling with the NimbleGen one-color DNA labeling kit; (step 3) array hybridization using the NimbleGen Hybridization System, followed by washing with the NimbleGen wash buffer kit; and (step 4) array scanning using the Axon GenePix 4000B microarray scanner (Molecular Devices Corporation). Scanned images were then imported into NimbleScan software (version 2.5) for grid alignment and expression data analysis. Expression data were normalized through quantile normalization and the Robust Multichip Average (RMA) algorithm included in the NimbleScan software. Probe-level and gene-level files were generated after normalization. All the gene-level files were imported into Agilent GeneSpring GX software (version 12.1) for further analysis. Differentially-expressed genes with statistical significance (p < 0.05) were identified through Volcano Plot filtering. Hierarchical clustering was performed to show distinguishable gene expression profiling among samples.

### Quantitative RT-PCR

Total RNA was extracted from cells using TRIzol reagent (Invitrogen) according to the manufacturer’s protocol. Then, reverse transcription was performed with the PrimeScript^®^ RT reagent kit (Takara), followed by real-time PCR using an ABI 7500 Sequence Detection System with a SYBR^®^ Premix Ex Taq™ kit (Takara). The sequences of the specific PCR primers were as follows:

AGT: Forward: 5′-GTACATACACCCCTTCCACCTCGTC-3′

Reverse: 5′-GTCAATCTTCTCAGCAGCAACATCC-3′

Renin: Forward: 5′-CACCAGCGCGGACTATGTAT-3′

Reverse: 5′-ACGCCGATCAAACTCTGTGT-3′

ACE: Forward: 5′-TGCTAGCCCTCTCAGTGTCT-3′

Reverse: 5′-CCACTGATCGACGAGGTAGC-3′

Chymase: Forward: 5′-GGAAATGTGAGCAGATAGCGCAGTC-3′

Reverse: 5′-AATCCGGAGCTGGAGAACTCTTGTC-3′

GAPDH: Forward: 5′- TGTCGTGGAGTCTACTGGTG-3′

Reverse: 5′-GCATTGCTGACAATCTTGAG-3′

GAPDH was used as the internal control.

### Construction of RNAi lentiviral vector and establishment of AGT-silenced cells

The designed shRNA construct contained a unique 19-nt double-stranded AGT target sequence that presented as an inverted complementary repeat, a loop sequence (5′-CTCGAG-3′), the RNA PloIII terminator (5′-TTTTTT-3′), and 5′ single-stranded overhangs for ligation into AgeI- and EcoRI-digested Pglv-u6-Puro lentivirus vector (GenePharma, Shanghai, China). The recombinant vector was named pGLV–AGT–shRNA. The shRNA human AGT-targeting sequence was 5′-GCATCCTCCTCGAACTCAA-3′. The negative control vector (pGLV–NC–shRNA) contained a nonsense shRNA insert with the sequence 5′-TTC TCCGAACGTGTCACGT-3′ in order to control any effects caused by non-RNAi mechanisms. We co-transfected the 293 T cells with three optimized packaging plasmids (pGag/Poll, pRev and pVSV-G) and the pGLV-AGT-shRNA or Pglv-NC-shRNA expression clone construct, which produced lentiviral stocks with a suitable titer. Stably transduced CNE2 and 5–8F cells were selected using puromycin, adding the minimum concentration of puromycin required to kill untransduced CNE2 or 5–8F cells. The efficiency of knockdown was detected by real-time qPCR and western blotting.

### Animal Models

All experimental procedures were approved and overseen by Southern Medical University Institutional Animal Care and Use Committee, and were performed in accordance with the guidelines and regulations for animal experiments set down by Southern Medical University. Four-week-old female BALB/C nude mice were used for all animal experiments. CNE2, 5–8F or MDA-MB-231 tumor cells (5 × 10^6^ cells) were inoculated s.c. into the right upper leg of each mouse. Tumors were allowed to grow for 8 days before randomization and treatment. To explore the influence of candesartan in radiosensitivity of tumors, the right upper leg tumor site was irradiated with 10 Gy on day 8 and candesartan (5 mg/kg, i.p., Takeda Pharmaceutical Company Limited, Japan) was given daily for 3 days before radiation and 3 days after radiation. Irradiation was performed at room temperature using 6-MV X-rays generated by a linear accelerator (Varian 2300EX; Varian, Palo Alto, CA). Tumors were measured every three days from the start of irradiation for 3 to 8 weeks, as indicated in the results. Tumor volume was calculated as length × width^2^ × 0.5.

### Statistical analyses

Data from experiments are presented as means ± SEM and analyzed using an independent-samples t-test or ANOVA. All statistical analyses were performed using SPSS 13.0 software (SPSS, Chicago, IL), and a probability level of 0.05 was chosen for statistical significance.

## Additional Information

**How to cite this article:** Xie, G. *et al*. Hypoxia-induced angiotensin II by the lactate-chymase-dependent mechanism mediates radioresistance of hypoxic tumor cells. *Sci. Rep.* **7**, 42396; doi: 10.1038/srep42396 (2017).

**Publisher's note:** Springer Nature remains neutral with regard to jurisdictional claims in published maps and institutional affiliations.

## Supplementary Material

Supplemental Information
