# Supplementary material for: Hypoxia-induced angiotensin II by the lactate-chymase-dependent mechanism mediates radioresistance of hypoxic tumor cells
Source: Sci Rep. 2017 Feb 16;7:42396. doi: 10.1038/srep42396 (PMC5311966; doi:10.1038/srep42396)
Supplement: Supplemental Information [file srep42396-s1.docx]

**Hypoxia-induced angiotensin II by the lactate-chymase-dependent mechanism** **mediates radioresistance of hypoxic tumor cells**

Guozhu Xie^*#1^, Ying Liu^*1^, Qiwei Yao^*3^, Zheng Rong^1^, Lanfang Zhang^1^, Jie Lin^1^, Zhaoze Guo^4^, Shasha Du^1^, Chen Ren^1^, Quan Yuan^5^, Yawei Yuan^#1,2^

^1^ Department of Radiation Oncology, Nanfang Hospital, Southern Medical University, Guangzhou, Guangdong 510515, PR China;

^2^ Department of Radiation Oncology, Cancer Center of Guangzhou Medical University, Guangzhou, Guangdong 510095, PR China;

^3^ Department of Radiation Oncology, Teaching Hospital of Fujian Provincial Cancer Hospital, Fuzhou, Fujian 350014, PR China;

^4^ Breast Center, Nanfang Hospital, Southern Medical University, Guangzhou, Guangdong 510515, PR China;

^5^ Jules Stein Eye Institute, David Geffen School of Medicine, University of California, Los Angeles, CA 90095, USA

^*^ Authors share co-first authorship.

^#^Authors share co-corresponding authorship.

**Supplementary Figures**

**
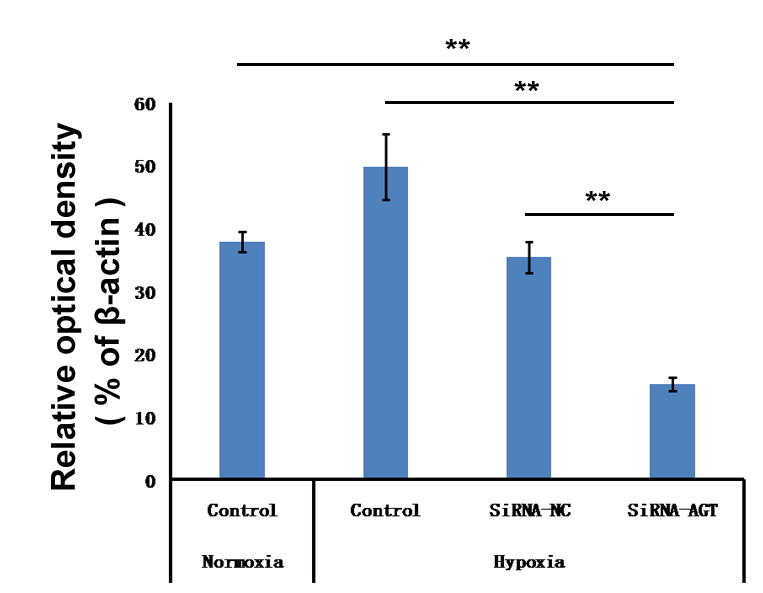
**

**Supplementary Figure 1 Relative optical density of western blotting in Figure 2a.**


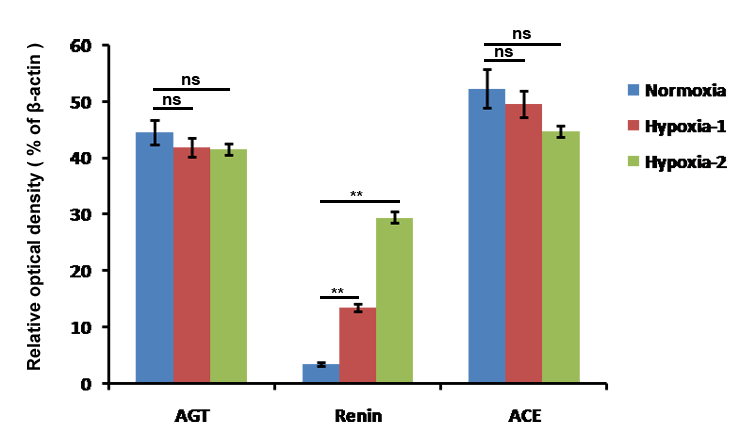


**Supplementary Figure 2 Relative optical density of western blotting in Figure 3b. Hypoxia-1, 5% O2 condition; hypoxia-2, 1% O2 condition.**


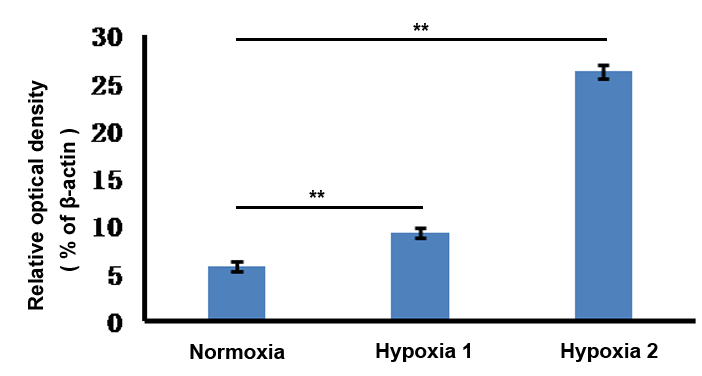


**Supplementary Figure 3. Relative optical density of western blotting in Figure 4c.**

**
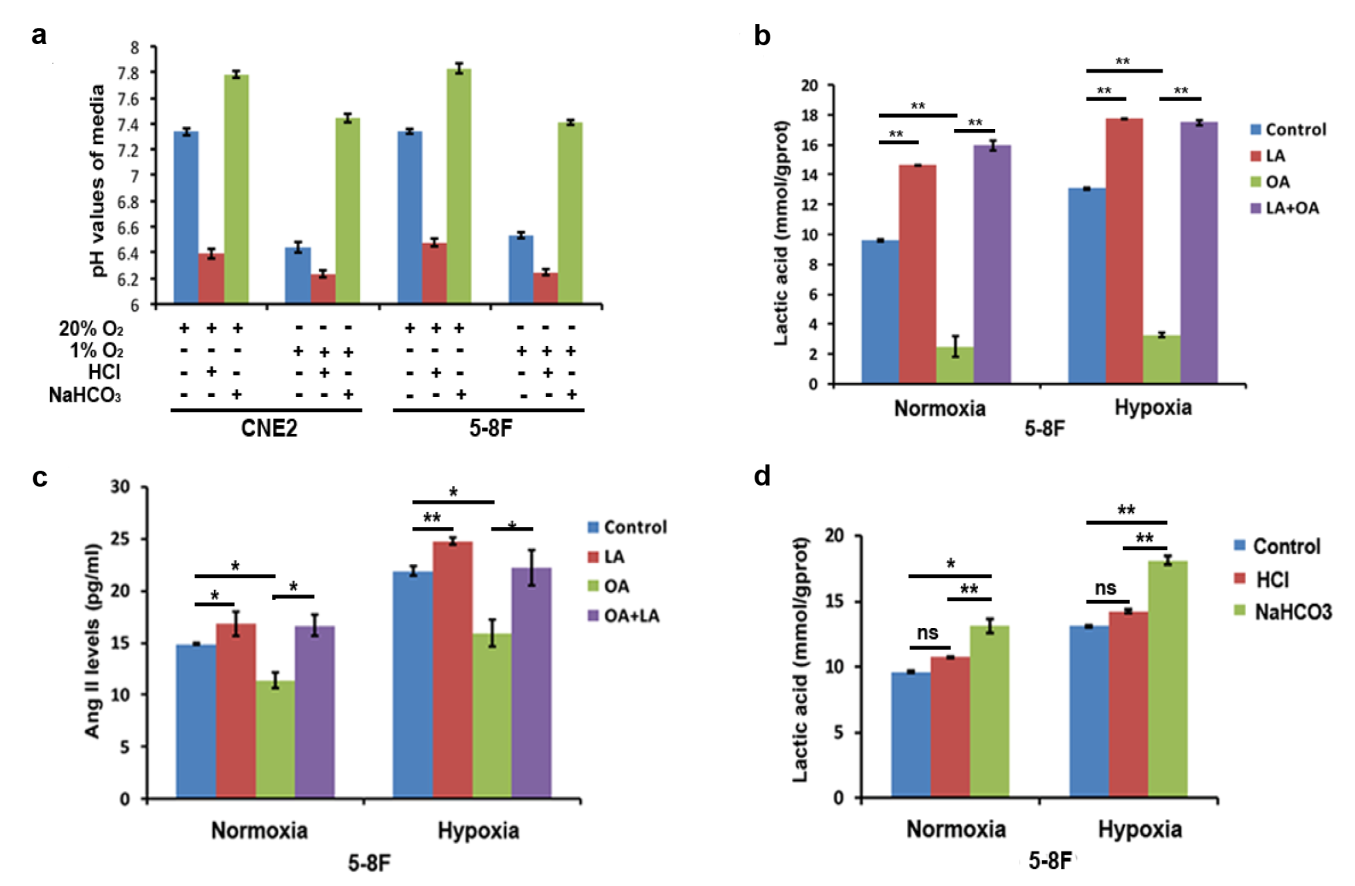
**

**Supplementary Figure 4** **The role of lactate or pH values on renin-chymase-mediated Ang II generation. (a)** The pH values of the conditioned medium from normoxic-cultured CNE2 and 5-8F cells were adjusted to the level of hypoxic-conditioned medium with 1% hydrochloric acid (HCl) , the pH value of the hypoxic-conditioned medium of CNE2 and 5-8F cells were adjusted to the level of normoxic-conditioned medium by adding 5% sodium bicarbonate (NaHCO_3_) during hypoxia. **(b)** The intracellular lactate levels were detected by a lactate assay after treatment with 5 mM lactate (LA) or 90 mM oxamic acid (OA) in normoxic or hypoxic 5-8F cells. **(c)** Ang II levels in conditioned medium were detected by ELISA after treatment with 5 mM lactate (LA) or 50 mM oxamic acid (OA) in normoxic or hypoxic 5-8F cells. **(d)** Adding NaHCO_3_ to medium of 5-8F cells during hypoxia or normoxia remarkably induced the increase of intracellular lactate levels, whereas the addition of HCl could not induce lactate product in normoxic or hypoxic conditions.


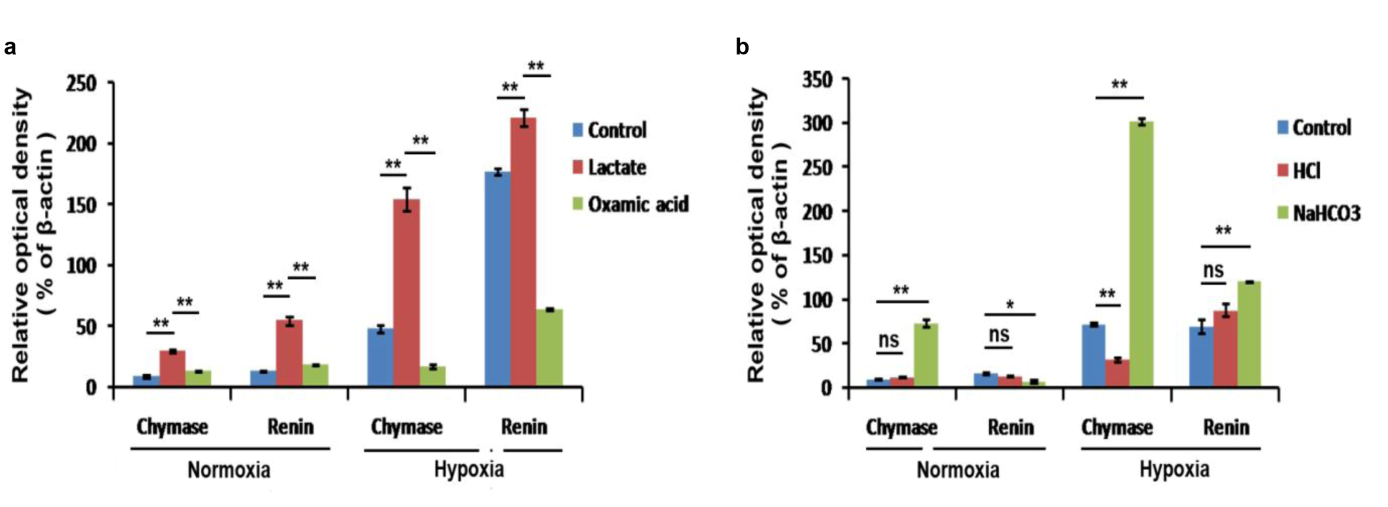


**Supplementary Figure 5 Relative optical density of western blotting in Figure 6.**


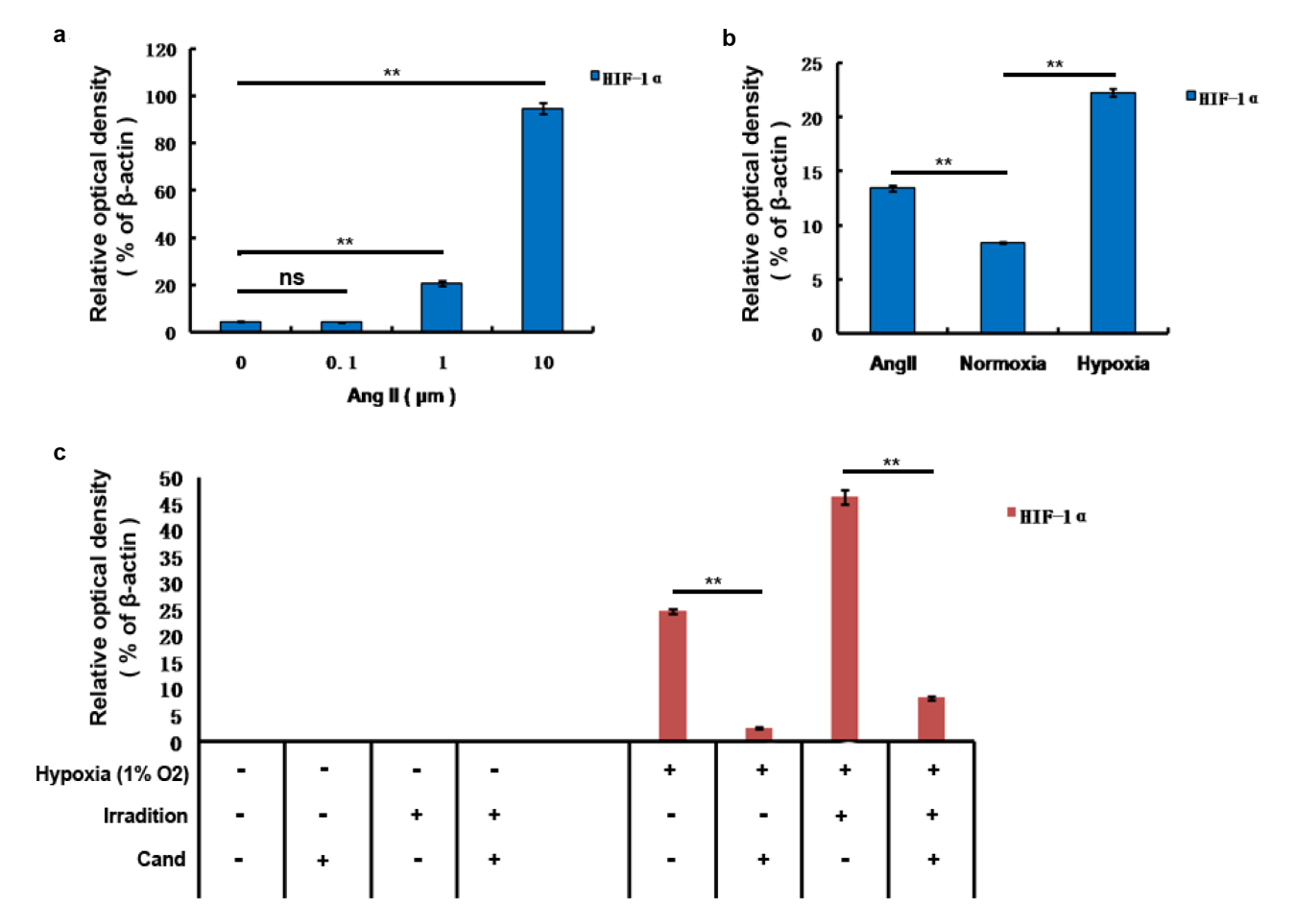


**Supplementary Figure 6 Relative optical density of western blotting in Figure 7.**


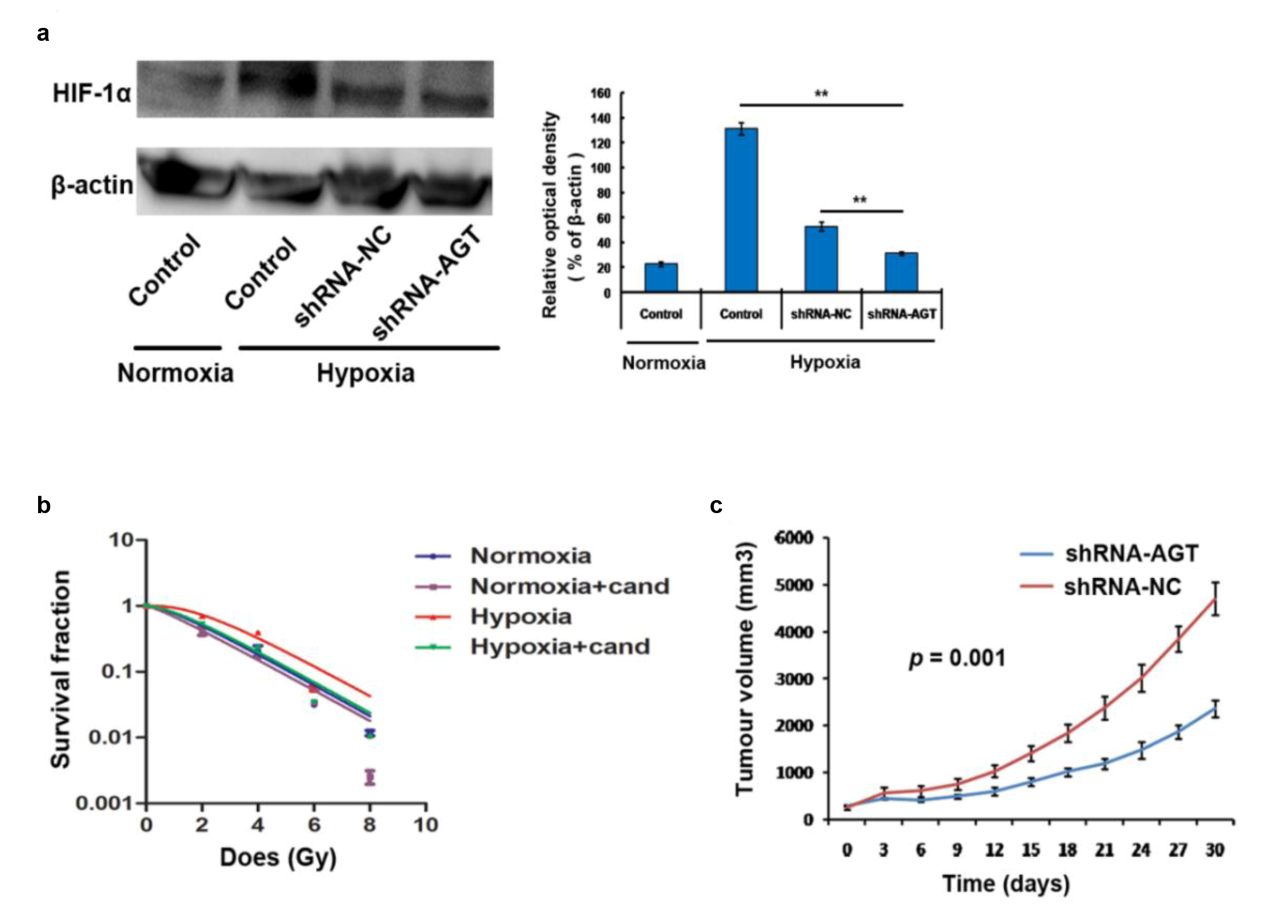


**Supplenentary Figure 7 Ang II signal is involved in the radiation resistance of tumor cells in the hypoxic condition. (a)** AGT-silence resulted in the reduced expression of HIF-1α protein in hypoxic CNE2 cells. **(b)** Hypoxia exposure markedly augmented the radioresistance of 5-8F cells, and the radioresistance of hypoxic 5-8F cells was greatly reversed if these tumor cells were pretreated with candesartan although candesartan did not significantly increase the radiosensitivity of normoxic 5-8F cells. **(c)** Tumors formed by AGT-silent 5-8F cells consistently reduced tumor volumes after 10-Gy irradiation as compared with their negative-control group.

**Supplementary Table 1. Antibodies for Immunofluorescence and Western Blotting**

| **Antigen** | **Cat. No.** | **Supplier** |
| --- | --- | --- |
| Angiotensin II | 251229 | Abbiotec |
| Angiotensinogen | Ab108334 | Abcam |
| Renin | Ab125012 | Abcam |
| ACE | Ab77990 | Abcam |
| Chymase | Ab186417 | Abcam |
| HIF-1α | 610958 | BD Bioscience |
| Pimonidazole | HP2-1000Kit | Hypoxyprobe Inc. |
